# The essential role of O-GlcNAcylation in hepatic differentiation

**DOI:** 10.1097/HC9.0000000000000283

**Published:** 2023-11-06

**Authors:** Dakota R. Robarts, Manasi Kotulkar, Diego Paine-Cabrera, Kaitlyn K. Venneman, John A. Hanover, Natasha E. Zachara, Chad Slawson, Udayan Apte

**Affiliations:** 1 Department of Pharmacology, Toxicology and Therapeutics, University of Kansas Medical Center, Kansas City, Kansas, USA; 2 Laboratory of Cell Biochemistry and Molecular Biology, NIDDK, NIH, Bethesda, Maryland, USA; 3 Department of Biological Chemistry, Johns Hopkins University School of Medicine, Baltimore, Maryland, USA; 4 Department of Biochemistry and Molecular Biology, University of Kansas Medical Center, Kansas City, Kansas, USA

## Abstract

**Background:**

O-GlcNAcylation is a post-translational modification catalyzed by the enzyme O-GlcNAc transferase, which transfers a single N-acetylglucosamine sugar from UDP-GlcNAc to the protein on serine and threonine residues on proteins. Another enzyme, O-GlcNAcase (OGA), removes this modification. O-GlcNAcylation plays an important role in pathophysiology. Here, we report that O-GlcNAcylation is essential for hepatocyte differentiation, and chronic loss results in fibrosis and HCC.

**Methods:**

Single-cell RNA-sequencing (RNA-seq) was used to investigate hepatocyte differentiation in hepatocyte-specific O-GlcNAc transferase-knockout (OGT-KO) mice with decreased hepatic O-GlcNAcylation and in O-GlcNAcase-KO mice with increased O-GlcNAcylation in hepatocytes. Patients HCC samples and the diethylnitrosamine-induced HCC model were used to investigate the effect of modulation of O-GlcNAcylation on the development of liver cancer.

**Results:**

Loss of hepatic O-GlcNAcylation resulted in disruption of liver zonation. Periportal hepatocytes were the most affected by loss of differentiation, characterized by dysregulation of glycogen storage and glucose production. O-GlcNAc transferase-KO mice exacerbated diethylnitrosamine-induced HCC development with increased inflammation, fibrosis, and YAP signaling. Consistently, O-GlcNAcase -KO mice with increased hepatic O-GlcNAcylation inhibited diethylnitrosamine-induced HCC. A progressive loss of O-GlcNAcylation was observed in patients with HCC.

**Conclusions:**

Our study shows that O-GlcNAcylation is a critical regulator of hepatic differentiation, and loss of O-GlcNAcylation promotes hepatocarcinogenesis. These data highlight increasing O-GlcNAcylation as a potential therapy in chronic liver diseases, including HCC.

## Graphical abstract

**Figure:**
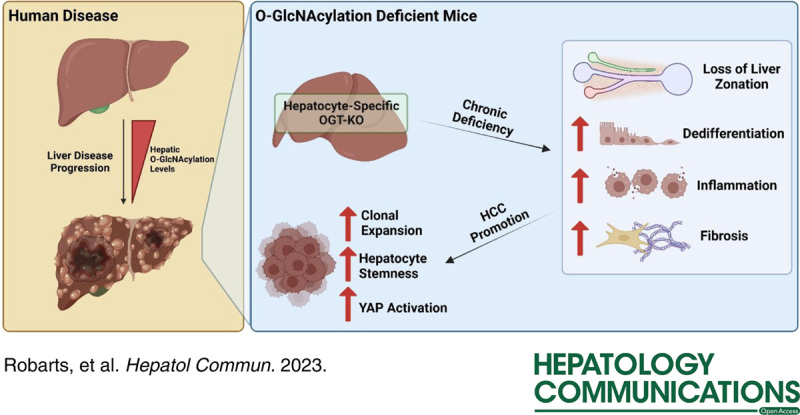

## INTRODUCTION

O-GlcNAcylation is a dynamic post-translational modification that involves the addition of N-acetylglucosamine (GlcNAc) onto proteins by means of an oxygen-linked bond. This process is regulated by two enzymes: O-GlcNAc transferase (OGT), which adds GlcNAc to proteins using UDP-GlcNAc as the GlcNAc donor, and O-GlcNAcase (OGA), which removes this modification.^1^ Because the hexosamine biosynthetic pathway, which produces UDP-GlcNAc, integrates multiple metabolic pathways, including nucleotide, fatty acid, protein, and glucose metabolism,^1^ changes in multiple independent metabolic pathways can affect O-GlcNAcylation. Studies show that O-GlcNAcylation plays a role in a number of cellular processes, including metabolism, inflammation, and cell proliferation.^1,2^ Abnormal O-GlcNAcylation levels have been linked to various diseases, including cancer.^1,3,4^ A recent study by our group found that a lack of hepatic O-GlcNAcylation during liver regeneration impairs the termination phase and leads to sustained cell proliferation and loss of hepatocyte (Heps) identity.^2^ Whereas O-GlcNAcylation is known to be involved in hepatic fibrosis and HCC pathogenesis, the mechanisms are not clear.^4–6^ These findings suggest that maintaining proper levels of O-GlcNAcylation may be important for the maintenance of healthy cells and the prevention of certain degenerative diseases.

During disease progression in the liver, hepatocyte nuclear factor 4 alpha (HNF4α), a critical regulator of maintaining Heps differentiation, is known to decrease, causing a decrease in liver function and Heps dedifferentiation.^7,8^ This leads to increased cell proliferation, ultimately increasing susceptibility to progression to HCC.^7,8^ O-GlcNAcylation is a critical regulator in cellular differentiation, such as hematopoietic stem cells and neuronal cells.^9,10^ Studies from our lab have shown cross-talk between O-GlcNAcylation and HNF4α.^2^ Here, we studied the effects of chronic loss of O-GlcNAcylation and the role of O-GlcNAcylation in the progression of liver disease. Using Heps-specific OGT knockout (KO) mice and single-cell RNA-sequencing (RNA-seq) technologies, we found that loss of O-GlcNAcylation leads to the loss of liver zonation, hyperplasic Heps nodules, and significant dedifferentiation. Further, we identified multiple molecular mechanisms at play in the regulation of hepatic differentiation by O-GlcNAcylation. Our data suggest that O-GlcNAcylation is a potential therapeutic target in the maintenance of hepatic differentiation during the progression of liver disease.

## METHODS

### Human liver samples

Human tissues were obtained from the University of Kansas Medical Center (KUMC) Liver Center (normal n of 4, NASH n of 3, NASH + cirrhosis n of 4). All human liver tissues were obtained with informed consent in accordance with the Declarations of Helsinki and Istanbul and ethical and institutional guidelines. All studies were approved by the Institutional Review Board of KUMC. HCC human tissue microarray was purchased from US Biolab (cat # LIV048-10A). The human protein atlas tool was used to visualize OGT protein levels in human HCC samples (tissue microarray #T-56000).^11^

### Animal care and models

The Institutional Animal Care and Use Committee at the University of Kansas Medical Center approved all animal studies that were performed in accordance with Institutional Animal Care and Use Committee regulations. All mice were housed in the KUMC vivarium with a standard 12-hour light and 12-hour dark cycle and on an ad libitum normal chow diet (LabDiet, cat# 5053). Generation of genetically altered OGT-floxed mice and OGA-floxed has been described.^12,13^ Both OGT-floxed and OGA-floxed were bred on a C57BL/6J background. Heps-specific KOs were generated by injecting AAV8-TBG (thyroxine-binding globulin)-CRE and using AAV8-TBG-eGFP (Vector Biolabs) as a control, as described.^2^

For chronic OGT deletion, 2-month-old male OGT-floxed mice were injected i.p. with AAV8-TBG-GFP or AAV8-TBG-CRE, and tissues were collected 35 days after AAV8 administration. To study liver cancer pathogenesis, OGT-floxed and OGA-floxed pups were treated with diethylnitrosamine (DEN, 15 μg/kg i.p.) on postnatal day 15. Five months after DEN injections, floxed mice were injected with AAV8-TBG-Cre to KO OGT or OGA. Controls were generated by injecting AAV8-TBG-EGFP. Mice were euthanized 2 months after AAV8 injection. In all studies, liver injury was assessed by serum alanine transaminase activity assay, and livers were weighed after cholecystectomy to calculate liver-weight-to-body-weight ratios as described.^2^ Glucose was measured in serum according to the manufacturer’s protocol (Pointe Scientific cat# G7521120).

### Staining procedures and imaging

Paraffin-embedded liver sections (5 µm thick) were used for hematoxylin and eosin staining, immunohistochemistry (IHC), and picrosirius red staining, as described.^2,14^ Primary and secondary antibodies with respective dilutions are shown in Supplemental Table S1, http://links.lww.com/HC9/A570. Photomicrographs were captured with an Olympus DP74 color camera mounted on an Olympus BX51 microscope with CellSens (Version 2.3) software.

### Protein isolation and western blotting

Approximately 100 mg of liver tissue was homogenized using a beaded tube containing 300 µL of Radio Immuno Precipitation Assay buffer (20 mM pH 7.5 Tris, 150 mM NaCl, 2 mM EDTA, 1 mM dithiothreitol, 40 mM GlcNAc, 0.1% sodium deoxycholate acid, 0.1% SDS, and 1%NP-40) containing 1x Halt Phosphatase inhibitor and protease cocktail (Thermofisher cat# 78427 & 78438). Pierce BCA Protein Assay (Thermofisher cat# 23225) was used to measure protein levels, as described.^15^ Antibodies used with respective dilutions are shown in Supplemental Table S1, http://links.lww.com/HC9/A570. All western blots were imaged with either SuperSignal West Pico PLUS or Femto Maximum (Thermofisher cat# 34578 and 34096). Western blots were imaged on Odyssey LiCor utilizing Image Studio software (Version 5.2).

### RNA Isolation and quantitative PCR

RNA was isolated from ~25 mg of liver tissue utilizing the TRIzol method according to the manufacturing protocol (Thermofisher cat# 15596026). Isolated RNA concentration was measured using an Implen N50 Nanophotometer. cDNA was made with 2000 ng of RNA per reaction utilizing the High-Capacity cDNA Reverse Transcription Kit according to the manufacturer’s protocol (Thermofisher cat# 4368814). Quantitative PCR (qPCR) was performed using 50 ng per reaction with PowerUp SYBR green master mix and a final concentration of 2.5 µM of forward and reverse primers (Supplemental Table S2, http://links.lww.com/HC9/A571) according to the manufacturer’s protocol (Thermofisher cat# A25741). A BioRad CFX384 system was used to run qPCR reactions in a 384-well plate setup. Raw data were analyzed using the CFX Maestro 1.1 Software (Version 4.1.2433.1219).

### Single-cell suspensions

Livers of chronic deleted OGT-KO mice (30 d) and controls were perfused utilizing 2-step collagenase perfusion to isolate parenchymal and nonparenchymal cells (NPC), as described.^16^ Trypan blue and a hemocytometer were used to determine cell viability and concentration of cells. All samples yielded a viability greater than 85%. A 50*g* centrifugation was used to pellet parenchymal cells from NPCs. Percoll gradients were used to remove dead cells. 100% buffered Percoll was made by a 1:9 ratio of 10x PBS to Percoll (Fisher Scientific cat# 45-001-747). Parenchymal cells were resuspended in 50% buffered Percoll and 50% isolation media [DMEM (Corning cat# 10014CV) supplemented with 10% fetal bovine serum and 2% bovine serum albumin] and centrifuged at 72*g* to remove dead cells. The parenchymal pellet was resuspended in isolation media to reach a concentration of 500 cells/µL. NPCs were pelleted using 600*g* centrifugation. Three milliliters of red blood cell lysis buffer (155 mM ammonium chloride, 10 mM potassium bicarbonate, and 0.1 mM EDTA, pH 7.3) was added to remove red blood cell contamination for 3 minutes. NPCs were then resuspended to a final concentration of 35.3% buffered Percoll and 64.7% isolation media and centrifuged at 900*g* for 10 minutes. The NPC pellet was then resuspended in isolation media for a concentration of 1200 cells/µL.

### Single-cell RNA-seq

Single-cell suspension of Heps and NPCs from OGT-KO and control mice was used for single-cell RNA-seq using the 10×Chromium Single-cell 3′ Gene Expression profiling platform targeting approximately 10,000 cells per sample and a read depth of 50,000 read per cell, as described in depth.^17^ Single-cell libraries were then sequenced using the Illumina NovaSeq 6000 S1 Flow Cell for 100 cycles. Raw data were analyzed utilizing 10x Cell Ranger using the mkfastq pipeline (Version 6.0.2) and aligned to the mouse transcriptome mm10-2020-A using the Apte Lab server (HPE DL380 Gen10 8SFF CTO high-performance server).^18^ Raw data were deposited into the Gene Expression Omnibus database (GSE223830). The aligned barcodes, features, and matrix files were uploaded into RStudio (Version 4.0.3, RStudio Team).

### Data analysis of single-cell RNA-seq

Data analysis was performed using the Seurat Package (Version 4.0.3). Single-cell data from parenchymal cells and NPCs for OGT-KO mice and controls were cleaned independently. For parenchymal cell analysis, duplicate cells or cells without a low read count and a high percentage of mitochondrial genes were kept in the analysis (if the number of captured features was between 100 and 7000, with mitochondrial genes being<75% of the features). The cell counts were then log normalized, scaled using a linear model, used the first 20 principal component analysis dimensions to determine nearest neighbors, and a resolution of 0.2 to generate clusters. Heps markers were identified using previously established markers, and all other cell types were filtered out.^17^ For the NPCs, cells were filtered using features between 100 and 7000, with mitochondrial genes being <20% then used a resolution of 0.4 for clustering. The markers within the Immunological Genome Project (ImmunoGen) database were used to identify populations within the NPC fraction. Heps were then filtered out of the NPCs. After data cleanup, the control NPCs were merged with OGT-KO NPCs and control Heps were merged with OGT-KO Heps to produce an NPC and Heps object. These 2 objects were then clustered using 0.2 resolutions for both the NPC and Heps. The cell types were then further annotated, including genotype labels. Finally, the merged Heps and NPC objects were merged and clustered using a 0.2 resolution. Cell contamination was exhibited between some clusters, likely due to a technical error, with cells clumping upstream of the sequencing. Each cell type was then subclustered to remove any residual contamination and reclustered to determine fine cell type labels using the ImmunoGen database. To predict cell-cell communication, we used the package CellChat (Version 1.5.0).^19^ The package standard workflow was used using the mouse interaction database.

### RNA-seq data acquisition

An online OGT data set in rodents was downloaded using SRA-tools from the Gene Expression Omnibus database (GSE188882).^2^ Raw fastq files were then aligned to the mouse genome (GRCm38) and counted using STAR software.^20^ DESeq2 (Version 1.28.1) in R Studio (Version 4.0.3, RStudio Team) was used for count normalization and differentially expressed gene lists, as described.^7^

### The Cancer Genome Atlas

The RStudio (Version 4.0.3, RStudio Team) package TCGAbiolinks (Version 2.16.4) was used to download liver HCC (The Cancer Genome Atlas-Liver Hepatocellular Carcinoma) RNA-seq data.^21^ The prebuilt EdgeR pipeline was used to generate differentially expressed gene lists with a log transformation. Analyzed data were annotated with biomaRT (Version 2.44.4) using the Ensembl database.

### Statistical analysis and data visualization

For experiments not associated with single-cell or bulk RNA-seq, such as alanine transaminase measurements, the results are expressed as mean ± SEM. Bar graphing and statistical analysis were carried out in GraphPad Prism 9. Student 2-tailed *t* test or 2-way ANOVA with Sidak post hoc test was applied to all analyses, with a *p*-value <0.05 considered significant. Dot plots, heatmaps, Venn diagrams, and uniform manifold approximation and projections were produced in RStudio (R version 4.0.3; RStudio Team).

## RESULTS

### O-GlcNAcylation is required to maintain liver homeostasis and contributes to the maintenance of hepatic zonation

**FIGURE 1:**
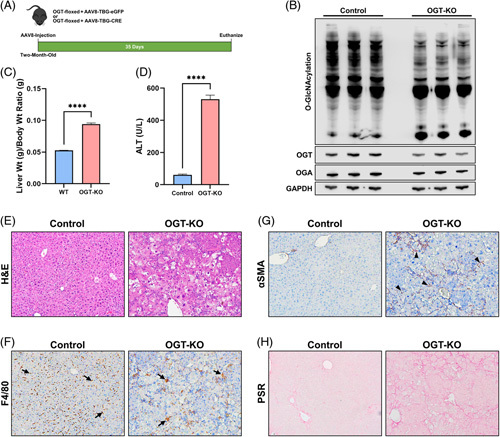
Twenty-eight-day deletion of OGT leads to dysplastic liver lobules. (A) Scheme of model for OGT 28-day deletion. (B) Western blot analysis of hepatic O-GlcNAcylation, OGT, OGA, and GAPDH from 35-day OGT-KO mice. (C) Liver weight-to-body weight ratios and (D) ALT serum levels of control and OGT-KO mice. The bar represents the mean, with error bars representing SEM. (E) Hematoxylin and eosin microsections of control and OGT-KO mice 35 days after deletion. Immunohistochemistry of the macrophage marker F4/80 (F) and the activated hepatic stellate cell marker αSMA in control and OGT-KO mice (×200 magnification) (G). (H) PSR staining to visualize collagen deposition in control and OGT-KO mice (×200 magnification). Arrow and arrowheads point to F4/80 and αSMA positive cells, respectively. Level of significance: ****p<0.0001 (2-tailed *t* test). Abbreviations: αSMA, alpha smooth muscle actin; GlcNAc, N-acetylglucosamine; H&E, hematoxylin and eosin; KO, knockout; OGA, O-GlcNAcase; OGT, O-GlcNAc transferase; PSR, Picrosirius red; WT, wild type.

To study the effects of a chronic loss of O-GlcNAcylation in Heps, we studied histopathological and molecular changes in Heps-specific OGT-KO mice 35 days after OGT deletion. Western blot analysis was used to confirm successful OGT-KO (Figure 1B). Similar to other studies, some OGT and O-GlcNAcylation were exhibited due to nonparenchymal cells retaining OGT expression.^2^ OGT-KO mice exhibited significant hepatomegaly, as indicated by an increase in liver-weight-to-body-weight ratio, and increased liver injury (Figure 1C, D). Hematoxylin and eosin staining of OGT-KO livers showed significant Heps dysplasia (Figure 1E), which was accompanied by reorganization of F4/80^+^ macrophages (Figure 1F), a significant increase in alpha smooth muscle actin, a marker for activated HSCs (Figure 1G). Picrosirius red staining revealed significant collagen deposition in OGT-KO livers (Figure 1H).

To interrogate changes in specific cell populations, we turned to single-cell RNA-seq (scRNA-seq). Unsupervised cell clustering produced 11 unique clusters identified as KCs, Heps, endothelial (Endo) cells, B cells, natural killer cells, T cells, dendritic cells, neutrophils, and monocytes in both control and OGT-KO mice (Supplemental Figure S1A–C, http://links.lww.com/HC9/A572). Specific markers were used to identify each cluster (Supplemental Figure S1D, http://links.lww.com/HC9/A572). T cells and Heps were the most captured cells in the control and OGT-KO mice, respectively. The least common were monocytes (control) and Endo cells (OGT-KO) (Supplemental Figure S1E, F, http://links.lww.com/HC9/A572). HSCs were not captured during the single-cell isolation.

**FIGURE 2:**
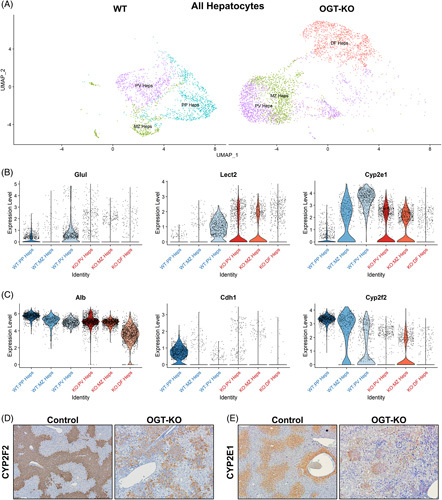
Single-cell RNA-seq analysis of hepatocytes derived from 35-day OGT-KO mice. (A) UMAP plot of SC RNA-seq of hepatocytes split by control (WT) and 35-day OGT-KO mice. Violin plots of (B) PV markers (*Glul*, *Lect2*, and *Cyp2e1*) and (C) PP markers (*Alb*, *Cdh1*, and *Cyp2f2*). Each black dot represents a hepatocyte with the distribution of the expression levels. Immunohistochemistry of CYP2F2 (D) and CYP2E1 (E) in control and OGT-KO livers (×200 magnification). Abbreviations: DF, dedifferentiated; GlcNAc, N-acetylglucosamine; Heps, hepatocytes; KO, knockout; MZ, midzonal; OGT, O-GlcNAc transferase; PP, periportal; PV, perivenous; RNA-seq, RNA-sequencing; UMAP, uniform manifold approximation and projection; WT, wild type.

The Heps exhibited the greatest difference at a single-cell resolution. The OGT-KO Heps exhibited minimal overlap with the control Heps (Figure 2A). We subclustered the Heps into either periportal (PP), midzonal (MZ), or perivenous (PV) Heps using the following gene markers: *Glul*, *Lect2*, and *Cyp2e1* for PV; *Alb*, *Cdh1*, and *Cyp2f2* for PP Heps. The control liver lobule had distinct PP and PV Heps populations, with MZ having an overlap of both PP and PV markers, particularly *Cyp2e1* and *Cyp2f2* (Figure 2B, C). The chronic deletion of OGT showed a loss of expression but retention of PV features with a lower percentage of cells expressing PV markers (Figure 2B, Supplemental Table S3, http://links.lww.com/HC9/A573) and complete loss of PP features (Figure 2C, Supplemental Table S3, http://links.lww.com/HC9/A573). An additional population that lacked both PP and PV features was identified in the OGT-KO mice, which we termed as dedifferentiated (DF) Heps. We confirmed the scRNA-seq data by performing IHC for CYP2F2 and CYP2E1 on control and OGT-KO liver sections. Consistent with scRNA-seq data, the control livers showed strong staining of CYP2F2 in the PP region and CYP2E1 in the PV region (Figure 2D, E), which was significantly lower in OGT-KO Heps (Figure 2D, E).

**FIGURE 3:**
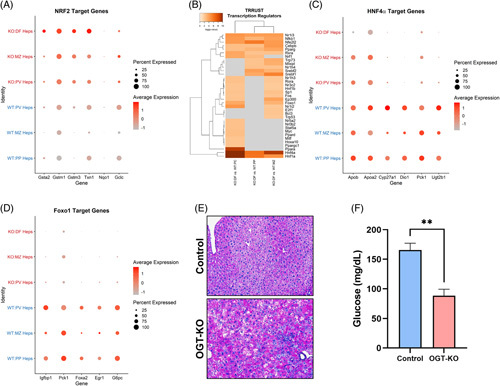
OGT-KO mice had decreased HNF4α activity and altered metabolic zonation of glycogen storage. (A) Dot plot of NRF2 target genes in each population represented in a dot plot. (B) Heatmap of the −log(*p*-value) for the altered TRRUST transcription regulators comparing the dedifferentiated hepatocytes to each control population. Gray represents no assigned *p*-value, and orange scale represents -log(*p*-value). Dot plot of (C) HNF4α and (D) FOXO1 target genes in each population, represented in a dot plot. (E) Periodic Schiff staining of livers of control and OGT-KO mice to visualize glycogen (×200 magnification). In the dot plots, the size represents the percentage of cells that express that gene, and the color represents the average expression in the cell population. (F) Bar graph of serum glucose levels in the control and OGT-KO mice. The bar represents the mean, with error bars representing SEM. Level of significance: **p<0.01 (2-tailed *t* test). Abbreviations: DF, dedifferentiated; GlcNAc, N-acetylglucosamine; Heps, hepatocytes; HNF4a, hepatocyte nuclear factor 4 alpha; KO, knockout; MZ, midzonal; OGT, O-GlcNAc transferase; PP, periportal; PV, perivenous; TRRUST, transcriptional regulatory relationships unravelled by sentence-based text-mining; WT, wild type.

Studies have shown that loss of O-GlcNAcylation results in increased NRF2 activity.^22,23^ Consistent with these findings, the Heps in the OGT-KO mice exhibited increased expression of the target genes *Gsta2*, *Gstm1*, *Txn1*, and *Gclc*, especially in the DF population (Figure 3A). To further characterize the DF population, we generated 3 differential gene lists of the comparisons KO:DF versus wild type:PP, versus wild type:PV, and versus wild type:MZ. Pathway analysis using Metascape was performed on these gene lists. HNF4α and HNF1α were the most significantly impacted regulators (Figure 3B). Target genes of HNF4α were accessed in all Heps populations and found decreased expression in *Apob*, *Apoa2*, *Cyp227a1*, *Dio1*, *Pck1*, and *Ugt2b1* in the OGT-KO populations compared to control. Notably, the DF Heps were the most affected compared to the OGT-KO MZ and PV populations (Figure 3C). FOXO1 was another impacted factor that is known to coregulate gene expression with HNF4α^24^ and is important in metabolic zonation of the liver, particularly glucose metabolism. To investigate this further, FOXO1 target genes were interrogated in the scRNA-seq data set. Interestingly, all 3 populations in the OGT-KO Heps showed a loss of FOXO1 activity (Figure 3D). To interrogate other methods of glucose metabolism, we performed Periodic acid-Schiff staining on control and OGT-KO mice. As expected, glycogen was concentrated in the PP region in the control mice, whereas in the OGT-KO mice, glycogen storage did not show zonation, and a number of Heps exhibited complete loss of glycogen throughout the liver lobule (Figure 3E). To determine whether excess storage was due to hyperglycemia, we measured the glucose levels in the serum and found a significant decrease in the OGT-KO, indicating a flux of serum glucose to glycogen in the liver (Figure 3F). These data indicate that O-GlcNAcylation is critical for maintaining liver lobule zonation through HNF4α.

The NPC populations were then subclustered to determine changes in the OGT-KO (Supplemental Figure S2A–E, http://links.lww.com/HC9/A574). The most striking differences of the NPCs were increasing infiltrating monocytes and KCs (Supplemental Figure S2B, http://links.lww.com/HC9/A574), the change in the CD4^+^ T-cell population and increased of CD8^+^ T cells (Supplemental Figure S2C, http://links.lww.com/HC9/A574), and an increase in Fcer2a^+^ B cells (Supplemental Figure S2D, http://links.lww.com/HC9/A574). Cell-cell communication analysis of the fine-labeled populations showed that the main signal transponders were Endo cells and the main signal receivers were CD8^+^ T cells (Supplemental Figure S2F, G, http://links.lww.com/HC9/A574). This indicates that Endo cells could play a role in T-cell recruitment in the OGT-KO livers.

### Depletion of O-GlcNAcylation promotes DEN-induced HCC

**FIGURE 4:**
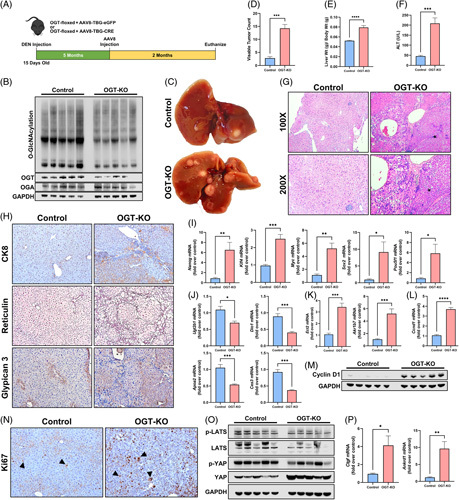
O-GlcNAcylation is an impediment to the progression of DEN-induced HCC. (A) Experimental design of DEN-induced HCC. (B) Western blot analysis of hepatic O-GlcNAcylation, OGT, and GAPDH in control and OGT-KO mice. (C) Gross photos of livers from control and OGT-KO after DEN-induced HCC. Bar graphs of visible tumor count (D), liver-weight-to-body-weight ratio (E), and serum ALT of control and OGT-KO mice (F). (G) Photomicrographs of hematoxylin and eosin of control and OGT-KO at ×100 and ×200 magnifications. (H) Tumor markers, including CK8, Reticulin, and Glypican 3, in control and OGT-KO mice (×200 magnification). Quantitative PCR (qPCR) of genes that regulate (I) hepatocyte stemness and hepatocyte nuclear factor 4 alpha upregulated (M) and downregulated target genes (N). (L) qPCR of cyclin D1, and (M) western blot analysis of the cell proliferation markers cyclin D1 and PCNA. (N) Immunohistochemistry of the proliferative marker Ki67 (200x magnification). (O) Western blot analysis of proteins involved in the YAP signaling pathway. (P) qPCR of YAP target genes. For qPCR, values were normalized to 18s then to the control group. Bars represent mean, with error bars representing the SEM. Arrow and arrowheads indicate dysplastic nodule and proliferating hepatocyte, respectively. Levels of significance: ****p<0.0001; ***p<0.001; **p<0.01; *p<0.05 (2-tailed t-test). Abbreviations: DEN, diethylnitrosamine; GlcNAc, N-acetylglucosamine; KO, knockout; OGA, O-GlcNAcase; OGT, O-GlcNAc transferase; PCNA, proliferating cell nuclear antigen; TBG, thyroxine-binding globulin.

Next, we investigated if loss of O-GlcNAcylation promotes the development of HCC using the DEN-induced HCC model.^25^ DEN was injected into OGT-floxed mice 15 days postnatal to initiate HCC development. OGT was deleted using the AAV8 system 5 months after DEN injection, and 2 months were allowed for tumor promotion (Figure 4A). Western blot analysis confirmed a decrease in total O-GlcNAcylation and OGT in OGT-KO mice compared to their control groups (Figure 4B). Importantly, OGT-KO mice had significantly more tumors compared to the control group (Figure 4C, D), with an increased liver-weight-to-body-wight ratio and liver injury (Figure 4E, F). Hematoxylin and eosin staining showed OGT-KO livers had significant neoplasia (Figure 4G), which was deemed to be HCC based on markers such as CK8, reticulin, and glypican 3 (Figure 4H). Because our studies show that O-GlcNAcylation is required for hepatic differentiation, we determined if there is increased stemness in OGT-KO livers with tumors. qPCR analysis showed a significant increase in stemness markers, including *Nanog*, *Klf4*, *Myc*, Sox2, and *Pou5f1* (OCT4 gene), in OGT-KO livers. During chronic liver disease, HNF4α function is known to decrease.^7^ We measured expression of genes regulated either positively (*Ugt2b1*, *Dio1*, *Apoa2*, and *Ces3*) or negatively (*Ect2* and *Akr1b7*) by HNF4α and found significant decrease and increase, respectively (Figure 4J, K).

**FIGURE 5:**
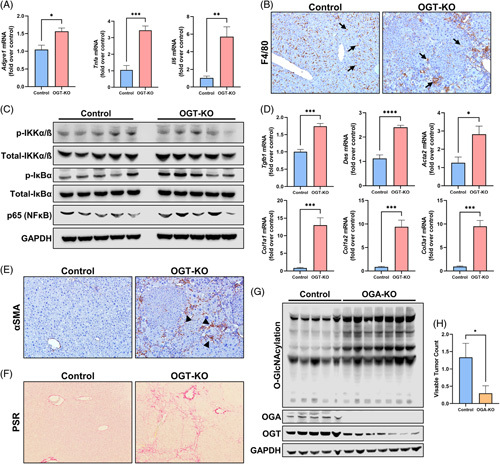
OGT-KO exhibited increased inflammation and fibrosis during the promotion of DEN-induced HCC. (A) qPCR of proinflammatory markers (*Adgre1*, *Tnfa*, and *Il6*). (B) Immunohistochemistry of F4/80. (C) Western blot analysis of NFkB pathway including phosphorylated/total Ikka/b, phosphorylated/total IkkBα, p65, and GAPDH. (D) qPCR of profibrotic genes (*Tgfb1*, *Des*, *Acta2*, *Col1a1*, *Col1a2*, and *Col3a1*). (E) Immunohistochemistry of αSMA and (F) picrosirius red staining in OGT-KO mice and controls. (G) Western blot analysis of total hepatic O-GlcNAcylation, OGT, and OGT from OGA-KO and controls treated with DEN. Bar graph for OGA-KO and control of (H) visible tumor counts. Photomicrographs are 200x magnification. Bars represent mean, with error bars indicating SEM. Level of significance: ****p<0.0001; ***p<0.001; **p<0.01; *p<0.05 (Two-tailed t-test). Abbreviations: αSMA, alpha smooth muscle actin; GlcNAc, N-acetylglucosamine; KO, knockout; OGA, O-GlcNAcase; OGT, O-GlcNAc transferase; PSR, Picrosirius red.

To determine the extent of cell proliferation, we measured the expression of cyclin D1 and found that it was significantly upregulated in OGT-KO mice (Figure 4L). This was corroborated by increased protein levels of cyclin D1, with a slight increase in proliferating cell nuclear antigen in OGT-KO mice (Figure 4M). IHC of Ki67 showed an increase in Heps proliferation, as well as NPC proliferation (Figure 4N). To investigate the mechanisms of increased DEN-induced carcinogenesis in OGT-KO mice, we investigated wingless protein, protein kinase B, and extracellular signal-regulated kinases signaling, and the Hippo Kinase pathway, all of which is known to be activated in HCC. Western blot analysis showed a significant decrease in extracellular signal-regulated kinases and protein kinase B activity (Supplemental Figure S3A, B, http://links.lww.com/HC9/A575). No changes were exhibited in either phosphorylated ß-catenin (inactive) or unphosphorylated ß-catenin (active) (Supplemental Figure S3C, http://links.lww.com/HC9/A575). qPCR of ß-catenin target genes showed no changes in *Axin2* and a significant suppression of *Cyp2e1* and *Glul* (Supplemental Figure S3D, http://links.lww.com/HC9/A575). Lastly, we found a decrease in phosphorylated LATS and phosphorylated Yap but an increase in total Yap in OGT-KO mice compared to control mice (Figure 4O). qPCR on YAP target genes (*Ctgf* and *Ankrd1*) corroborated the YAP activity data (Figure 4P). These data indicated that proliferation is governed by YAP signaling. OGT-KO mice exhibited a significant induction in the proinflammatory markers *Adgre1*, *Tnfa*, and *Il6*, inflammatory nodules, and NFκB signaling (Figure 5A–C). qPCR on profibrotic genes (*Tfgb1*, *Des*, *Acta2*, *Col1a1*, *Col1a2*, and *Col1a3*) was performed and found a significant induction in OGT-KO mice, which was corroborated by alpha smooth muscle actin IHC and Picrosirius red staining (Figure 5D–F).

To determine the effect of increased O-GlcNAcylation on HCC progression, we repeated the experiments in OGA-floxed mice (Figure 5G and Supplemental Figure S4A, http://links.lww.com/HC9/A576). We observed a striking reduction in visible tumors in the DEN-treated OGA-KO mice (Supplemental Figure S4B, http://links.lww.com/HC9/A576). However, there was no significant difference in liver-weight-to-body ratio, liver injury, histological changes, HCC markers, or cell proliferation between OGA-KO and control mice treated with DEN (Figure 5H and Supplemental Figure S4C–K, http://links.lww.com/HC9/A576). Additionally, OGA-KO mice did not have changes in inflammation or fibrosis (Supplemental Figure S5A–D, http://links.lww.com/HC9/A577).

### Hepatic O-GlcNAcylation levels decrease in chronic liver disease progression in humans

**FIGURE 6:**
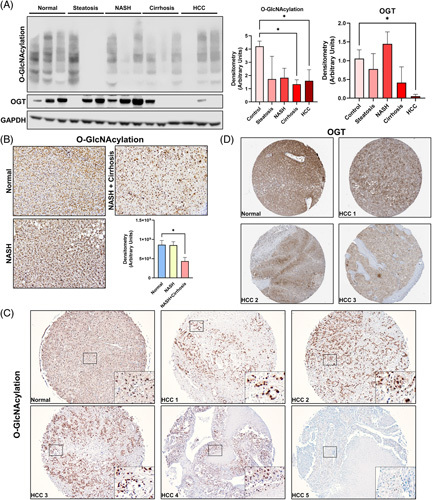
O-GlcNAcylation is lost during the progression of liver disease. (A) Western blot analysis of human liver samples for total O-GlcNAcylation, OGT, and the housekeeping protein GAPDH with corresponding densitometry. (B) Immunohistochemistry of total O-GlcNAcylation of livers from healthy, NASH, and NASH with cirrhosis human liver tissue. Immunohistochemistry of O-GlcNAcylation of HCC and normal human samples derived from tissue microarray (C) and OGT derived from the human protein atlas (D). Levels of significance: *p<0.05 (ANOVA). Abbreviations: GlcNAc, N-acetylglucosamine; OGT, O-GlcNAc transferase.

Western blot analysis using human liver samples of normal, steatosis, NASH, cirrhosis, and HCC showed a progressive decline in total O-GlcNAcylation (Figure 6A). Cirrhosis and HCC were significantly less than the controls whereas steatosis and NASH trended to be less. There was no difference in OGT protein levels in healthy, steatosis, and NASH samples, but OGT was significantly decreased in HCC samples compared to controls. IHC of O-GlcNAcylation in human liver samples of NASH or NASH + cirrhosis corroborated that O-GlcNAcylation is maintained in NASH without cirrhosis but is decreased in NASH with cirrhosis (Figure 6B). IHC of O-GlcNAcylation on tissue microarrays containing normal and HCC samples showed decreased O-GlcNAcylation and OGT in HCC (Figure 6C, D). Taken together, these data indicate that O-GlcNAcylation is lost during late-stage liver disease.

## DISCUSSION

O-GlcNAcylation is involved in a plethora of cellular processes, such as metabolism, proliferation, and cell differentiation.^1^ Because of its critical role in cellular functions, dysregulation of O-GlcNAcylation is known to be involved in diseased states, such as inflammation and fibrosis, both of which are hallmarks of HCC. In this study, we found that Heps-specific OGT-KO resulted in a loss of Heps differentiation and disruption in metabolic liver zonation. Further, we found that Heps-specific loss of OGT results in promotion of carcinogen-induced HCC, while OGA-KO mice, with higher hepatic O-GlcNAcylation, are protected from HCC development.

Studies have found that increased O-GlcNAcylation is a driver of HCC progression.^6,26–29^ Because our data contradict the hypothesis that increased O-GlcNAcylation is a promoter of HCC, we repeated DEN-induced HCC in OGA-KO mice. Interestingly, we found that increased hepatic O-GlcNAcylation had fewer visible tumors compared to their respective controls, indicating protection against HCC progression. However, no differences were exhibited in cell proliferation markers compared to the control group. The HCC model we implied was only 7 months after DEN injections with only 2 months of OGA deletion. This is a relatively short period to allow HCC to develop. We suspect that this was the reason the OGA-KO lacked significant changes in cell proliferation markers. Future studies should be done to promote the expansion of HCC in OGA-KO and control mice and using longer timepoints to determine the extent of HCC progression by increasing O-GlcNAcylation.^30^

Single-cell technologies have become more widely used to study the liver in different states.^17,31–33^ It is well established that the liver is metabolically zonated, particularly Heps, which show distinct molecular patterns linked to their metabolic function.^34^ To classify these Heps, markers for specific zones, either more PV- or PP-like Heps, have been determined.^17,35^ We used single-cell technologies to interrogate the effect of a chronic 5-week depletion of O-GlcNAcylation in Heps. At a single-cell level, very few Heps overlapped (between control and OGT-KO) using unsupervised clustering methods, indicating substantial transcriptome changes. This high-resolution sequencing data illustrate that not only O-GlcNAcylation affect Heps differentiation, but it also affected PP Heps at a greater magnitude compared to PV Heps at the transcriptional level. This is likely attributed to the loss of HNF4α in OGT-KO Heps populations. It is well established that loss of HNF4α leads to the dedifferentiation of Heps into hepatoblast-like cells, allowing them to be in a more proliferative state.^36^ Past studies from our lab showed that HNF4α levels need to decrease for Heps to proliferate and chronic lack of HNF4α leads to liver disease progression.^7,36,37^ Interestingly, after two-third partial hepatectomy in OGT-KO mice, HNF4α levels are lost during liver regeneration, leading to a DF phenotype.^2^ HNF4α is known to upregulate PP genes while suppressing PV features in PP populations.^38^ Conversely, in the PV region, HNF4α is suppressed by LEF1, a WNT-regulated gene, to prevent PP features. A functional example is that HNF4α and FOXO1 are known to coregulate glycogen metabolism, which is metabolically zonated.^24^ Our data show that glycogen storage is no longer localized in the PP region but exists throughout the liver lobule. These data illustrate that O-GlcNAcylation is critical in the maintenance of hepatic differentiation and metabolic zonation by maintaining HNF4α levels.

Additionally, HNF4α is a key event in the development of HCC.^7^ HCC often manifests as a degenerative phenotype, becomes more severe, and eventually leads to liver failure.^7^ The pathogenesis of HCC is complex and varies among individuals. One mechanism that we propose that contributes to the development of HCC is the loss of O-GlcNAcylation, which leads to the loss of HNF4α. Our data are consistent with studies in which people with cirrhosis had decreased O-GlcNAcylation compared to healthy individuals.^5^ This is further sustained in those who develop HCC. However, other studies have shown that O-GlcNAcylation levels can be diverse in people with HCC, suggesting that both increased and decreased levels of O-GlcNAcylation may contribute to the development of HCC in individuals with liver disease.^39^

Interesting, we do see a discrepancy between the relationship between OGT and O-GlcNAcylation in human liver disease progression. The intricate control of O-GlcNAcylation extends beyond the mere modulation of OGT and OGA enzyme levels. We propose that this discrepancy can be attributed to the intracellular concentrations of UDP-GlcNAc. The regulation of UDP-GlcNAc is intricately influenced by the concentrations of metabolites involved in diverse pathways, such as glucose, nucleotide, fatty acid, and protein metabolism.^40^ Consequently, alterations in the levels of these metabolites have the potential to perturb UDP-GlcNAc concentrations, thereby instigating global changes in O-GlcNAcylation.^41^

Studies on the role of O-GlcNAcylation have used either *in vitro* ^3,4,39^ or xenograft models.^3,39^ Our data obtained using cell-specific OGT and OGA-KO *in vivo* illustrate that the lack of hepatic O-GlcNAcylation was more severe than increased O-GlcNAcylation. Decreased O-GlcNAcylation led to significant induction in the progression of HCC. Our data show that OGT-KO mice have significant injury, inflammation, fibrosis, disruption of metabolic zonation, and DF Heps, all of which are exhibited in HCC progression.^38,42,43^ This could be explained by a multitude of factors. Models knocking out OGT in Heps and biliary cells exhibit an induction of necroptosis, causing liver injury and inflammation.^5^ Additionally, O-GlcNAcylation is found to regulate serum response factor, which leads to the activation of HSCs and fibrosis.^44^ Additionally, our data corroborate other studies showing that O-GlcNAcylation is a critical regulator of cell proliferation.^2,45–47^ Further, our data exhibited increased stemness gene expression, indicating a more stem cell–like identity. HNF4α, hepatic master regulator, activity was also significantly downregulated. Both are signs of cell proliferation potential. Multiple pathways in the liver can govern cell proliferation. We found increased activity of YAP signaling, indicating the primary driver of proliferation, with no inductions in β-catenin, extracellular signal-regulated kinases, and protein kinase B signaling. The YAP regulation of O-GlcNAcylation is controversial. Two O-GlcNAcylation sites have currently been mapped to YAP, serine (Ser)127, and threonine (Thr)241. Interestingly, one site is thought to inhibit HCC progress (Ser127), while the other enhances disease progression (Thr241).^3,48^ Both modifications act by increasing the translocation and activity of YAP; however, each seems to have a different role. Ser127 leads to increased cell proliferation and survival, whereas Thr241 modification allows YAP to upregulate the transferrin receptor, promoting cell death through ferroptosis. One possible explanation for why it contributes to cell proliferation in our models is that HNF4α and YAP activities are intertwined.^49–51^ One mechanism proposed is that HNF4α competes with YAP in heterodimerization with TEAD4, inhibiting YAP activity.^51^ This indicates that a lack of HNF4α, triggered due to lack of O-GlcNAcylation, would cause an induction of YAP activity, leading to HCC progression.

In summary, these data show that the loss of O-GlcNAcylation is critical in maintaining hepatic differentiation and liver zonation. Loss of hepatic differentiation and increased cell death further promote inflammation and fibrosis, ultimately promoting HCC progression. While increasing hepatic O-GlcNAcylation had no effect on hepatic differentiation or HCC promotion. These data indicate that increasing O-GlcNAcylation could be a novel therapeutic strategy for chronic liver diseases, especially HCC.

## Supplementary Material

SUPPLEMENTARY MATERIAL
