# Supplementary material for: The essential role of O-GlcNAcylation in hepatic differentiation
Source: Hepatol Commun. 2023 Nov 6;7(11):e0283. doi: 10.1097/HC9.0000000000000283 (PMC10629742; doi:10.1097/HC9.0000000000000283)
Supplement: SUPPLEMENTARY MATERIAL [file hc9-7-e0283-s003.docx]

**Robarts et al,**

**Supplementary Materials**

**Hepatology Communications**

**
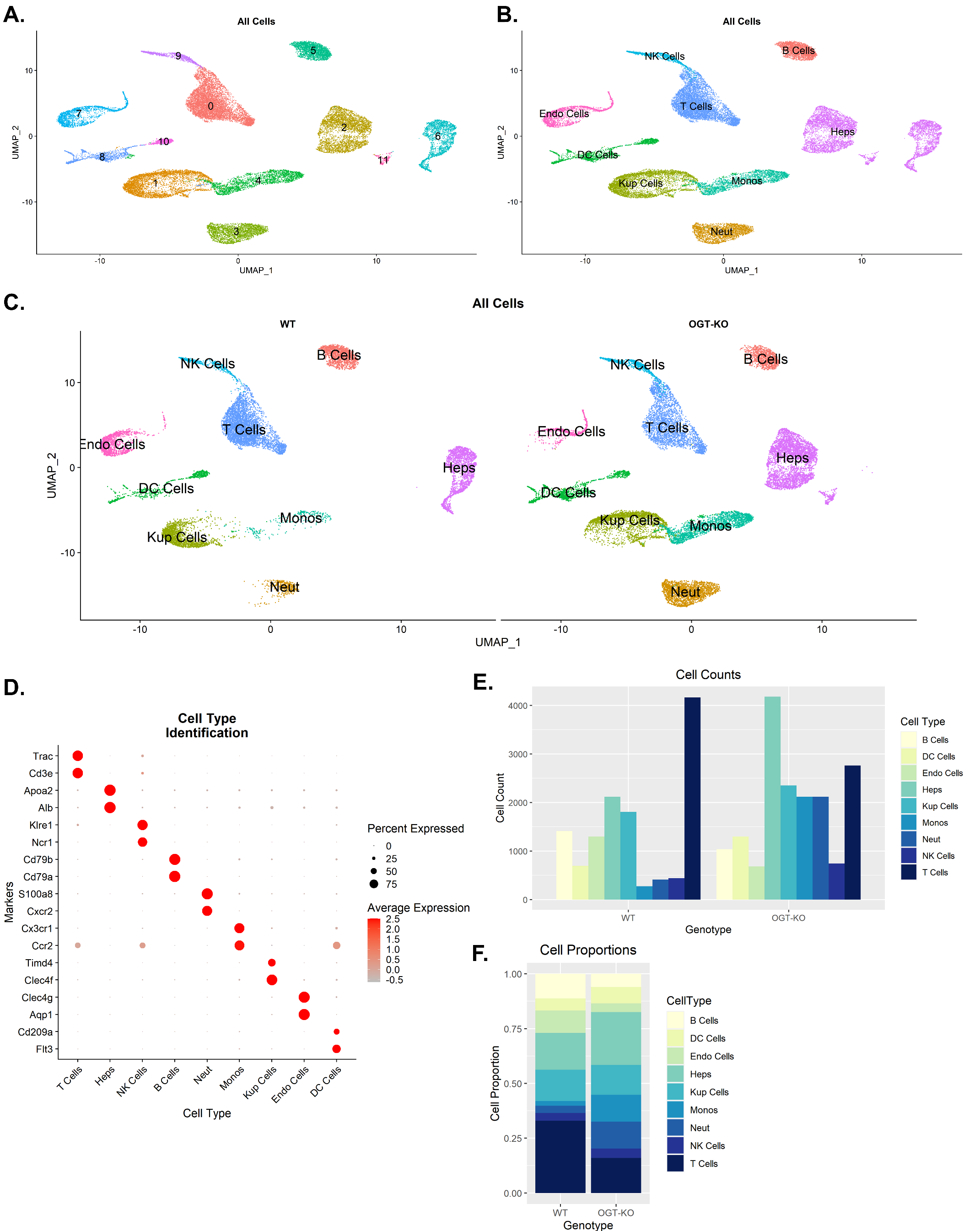
**

## **Figure S1. Single-cell RNA-sequencing cell type identification.**

UMAP of unsupervised clustering with (A) cluster identification number and (B) annotated clusters for OGT-KO and control samples. (C) A split UMAP of annotated clusters between control and OGT-KO. (D) Dot plot showing expression levels and percentage of population expressing two representative markers, which was used to annotate each cell type. Bar graph of (E) total number of cells in each population with (F) respective proportions.
