# Supplementary material for: The essential role of O-GlcNAcylation in hepatic differentiation
Source: Hepatol Commun. 2023 Nov 6;7(11):e0283. doi: 10.1097/HC9.0000000000000283 (PMC10629742; doi:10.1097/HC9.0000000000000283)
Supplement: SUPPLEMENTARY MATERIAL [file hc9-7-e0283-s005.docx]

**Robarts et al,**

**Supplementary Materials**

**Hepatology Communications**


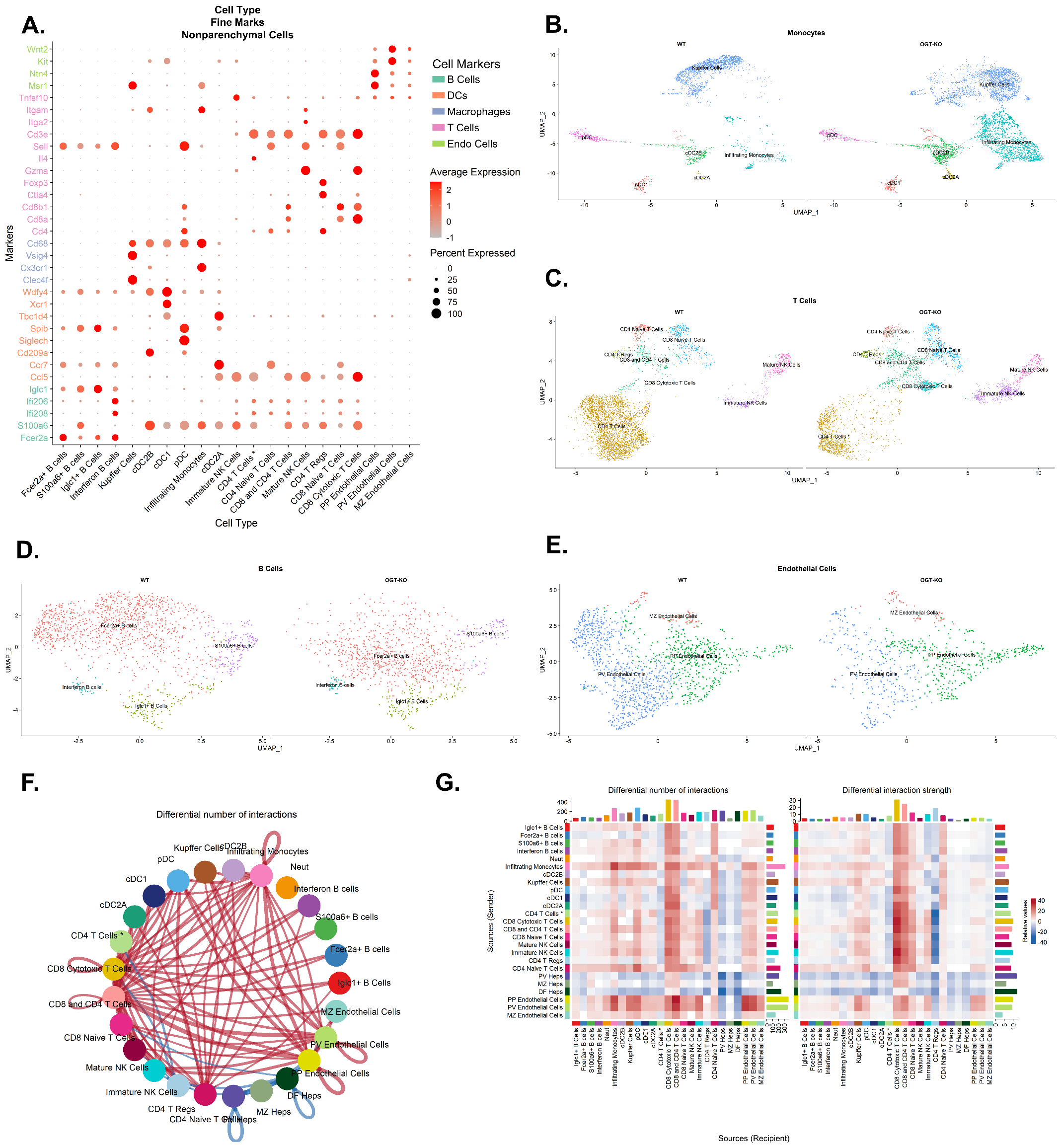


## **Figure S2. OGT-KO NPC populations significantly changed in the OGT-KO mice.**

(A) Dot plot of fine cell-type markers. The color of the dot represents the expression level, and the size of the dot represents the percentage of cells expressing the marker. Gene name color represents the larger category of cell types. UMAP of subcluster with fine labels of (B) monocytes, (C) t-cells, (D) B-cells, and (E) endothelial cells. (F) Cord diagram of predictive interaction between populations defined by fine cell type. Red and blue connections represent increased and decreased signaling in OGT-KO, respectively. Size of the connection indicates the number of cell-cell interactions. (G) Heatmap of differential number of interactions and interaction strength. Color scale represents increased (red) and decreased (blue) signals in OGT-KO mice compared to the control. Side bar graphs represent the sum of outgoing signals for the number and strength of interactions per cell type. The top columns represent the sum of incoming signals for the number and strength of interactions per cell type.
