# Supplementary material for: The essential role of O-GlcNAcylation in hepatic differentiation
Source: Hepatol Commun. 2023 Nov 6;7(11):e0283. doi: 10.1097/HC9.0000000000000283 (PMC10629742; doi:10.1097/HC9.0000000000000283)
Supplement: SUPPLEMENTARY MATERIAL [file hc9-7-e0283-s006.docx]

**Robarts et al,**

**Supplementary Materials**

**Hepatology Communications**

**
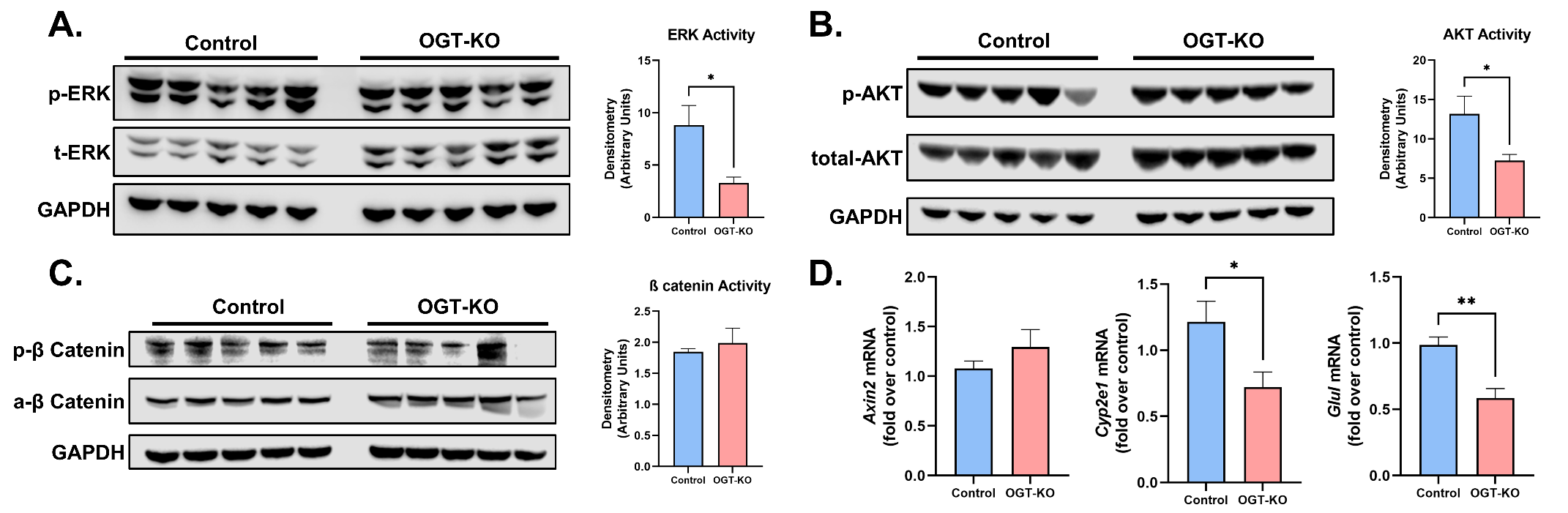
**

## **Figure S3. ERK, AKT, p38 and ß-catenin did not contribute to cell proliferation after DEN-induced HCC in OGT-KO mice.**

Western blot of (A) phosphorylated ERK and total ERK, (B) phosphorylated AKT and total AKT, and (C) phosphorylated ß-catenin and active ß-catenin (non-phosphorylated) with their respective quantification of activity. (D) qPCR of ß-catenin target genes normalized to 18s. Bars represent mean with error bars SEM. Level of significance: **p < 0.01; *p < 0.05 (Two-tailed t-test)
