# Supplementary material for: The essential role of O-GlcNAcylation in hepatic differentiation
Source: Hepatol Commun. 2023 Nov 6;7(11):e0283. doi: 10.1097/HC9.0000000000000283 (PMC10629742; doi:10.1097/HC9.0000000000000283)
Supplement: SUPPLEMENTARY MATERIAL [file hc9-7-e0283-s007.docx]

**Robarts et al,**

**Supplementary Materials**

**Hepatology Communications**


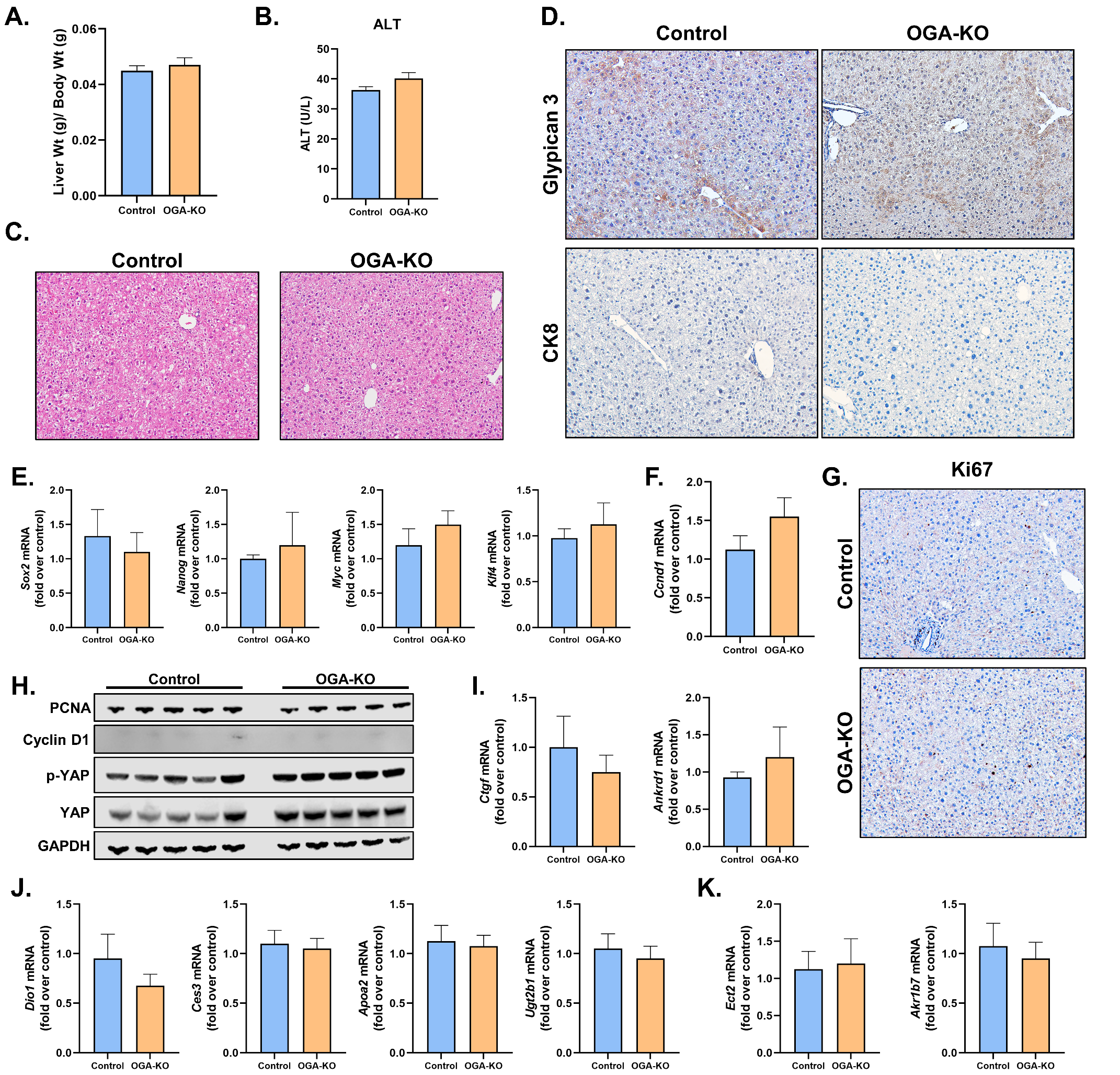


## **Figure S4. OGA-KO mice showed no significant changes in dedifferentiation and cell proliferation after DEN-induced HCC.**

Bar graphs for (A) liver-weight-to-body-weight ratio and (B) serum ALT levels. Photomicrographs of (C) H&E and (D) IHC of HCC markers Glypican 3 and CK8. qPCR of (E) stemness markers (*Sox2*, *Nanog*, *Myc*, and *Klf4*) and (F) *Ccnd1*. (G) IHC for the cell proliferation marker Ki67. (H) Western blot analysis of cell proliferation markers PCNA, cyclin D1, YAP, and phosphorylated yap. qPCR of (I) YAP target genes (*Ctgf* and *Ankrd1*) and gene (J) positively (*Dio1*, *Ces3*, *Apoa2*, and *Ugt2b1*) and (K) negatively (*Ect2* and *Akr1b7*) regulated by HNF4α. In bar graphs, the bar represents the mean and error bars SEM. Level of significance: *p < 0.05 (Two-tailed t-test)
