# Supplementary material for: The essential role of O-GlcNAcylation in hepatic differentiation
Source: Hepatol Commun. 2023 Nov 6;7(11):e0283. doi: 10.1097/HC9.0000000000000283 (PMC10629742; doi:10.1097/HC9.0000000000000283)
Supplement: SUPPLEMENTARY MATERIAL [file hc9-7-e0283-s008.docx]

**Robarts et al,**

**Supplementary Materials**

**Hepatology Communications**


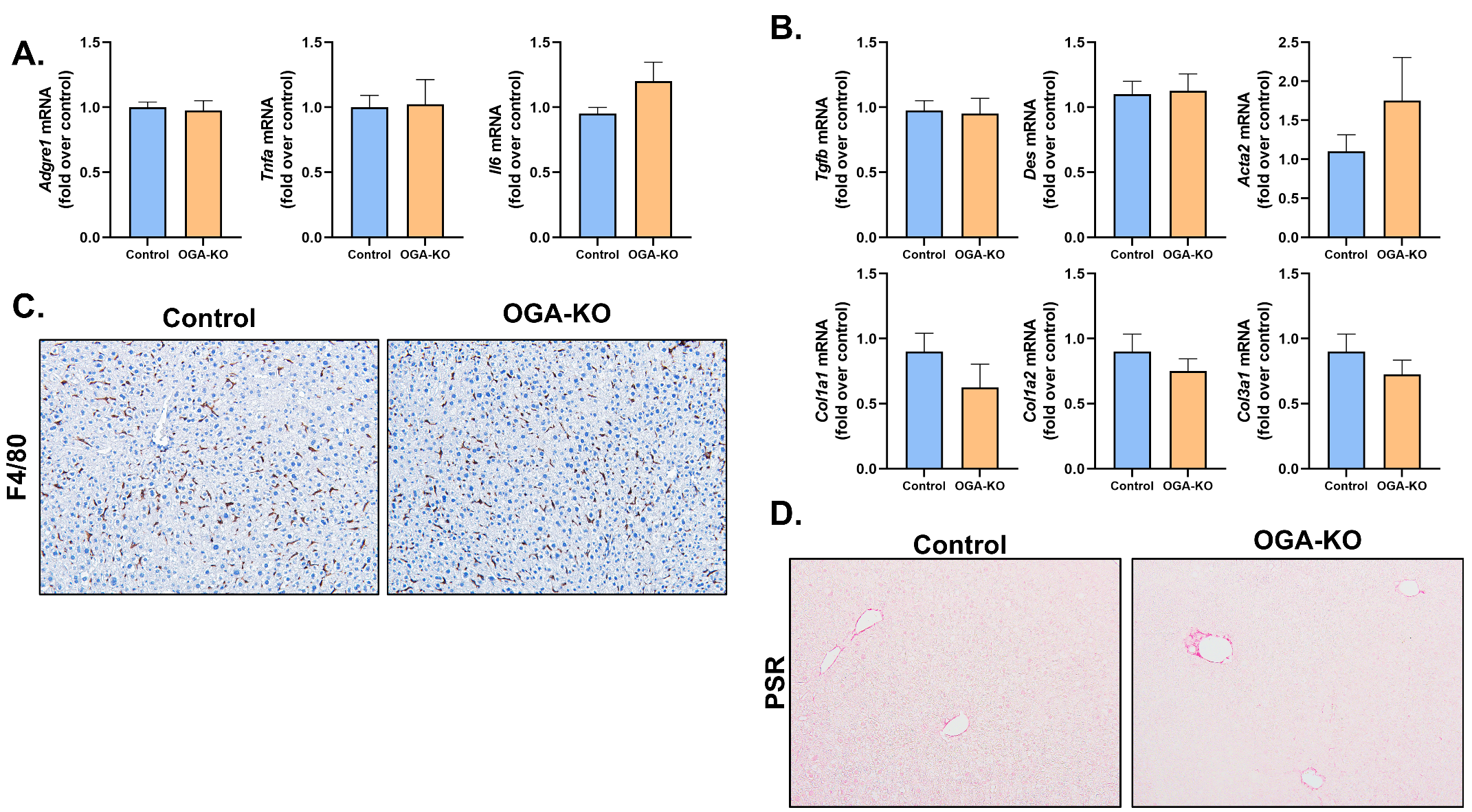


## **Figure S5. Increased O-GlcNAcylation led to no significant changes in inflammation or fibrosis that were exhibited after DEN-induced HCC.**

qPCR of (A) proinflammatory markers (*Adgre1*, *Tnfa*, and *Il6*) and (B) profibrotic genes (*Tgfb1*, *Des*, *Acta2*, *Col1a1*, *Col1a2*, and *Col3a1*). Bars represent the mean, with error bars meaning SEM. (C) IHC of the macrophage marker F4/80. (D) Picrosirius red staining in the OGA-KO and control mice. Level of significance: (Two-tailed t-test)
